# From genetic risk to early detection - clinical outcomes of a person-centered screening program for women with a high genetic risk of breast cancer

**DOI:** 10.3389/fonc.2025.1730423

**Published:** 2026-02-02

**Authors:** Ke Zhou, Caroline Abadie, Louise Crivelli, Euriell Fortin, Martine Bellanger, Charlotte Huet

**Affiliations:** 1 Departement of Human and Social Sciences, Institut de Cancérologie de l’Ouest, Saint Herblain, France; 2 Departement of Oncogenetics, Institut de Cancérologie de l’Ouest, Saint Herblain, France; 3 Departement of Oncogenetics, Centre Eugène Marquis, Rennes, France; 4 Coordination of the Phare Grand Ouest, Centre Eugène Marquis, Rennes, France

**Keywords:** *BRCA1*, *BRCA2*, breast cancer, clinical outcomes, early detection, genetic testing, germline pathogenic variant, screening

## Abstract

**Background:**

There is little evidence on breast cancer (BC) diagnosed in women with a high genetic risk, before and after their inclusion in a long-term risk management program based on genetic risk assessment. We analyzed clinical outcomes in women enrolled in the Phare Grand Ouest (PGO) program.

**Methods:**

The PGO includes carriers of the *BRCA1* and *BRCA2* pathogenic variants (PV) and women at high risk without *BRCA PV*, enrolled in eight cancer genetics units. The study population included all women with incident or prevalent BC, and 1:1 matching by age at first diagnosis was conducted. Multivariable generalized linear and logistic regression models were used to examine the associations between tumor size and cancer stage and the following covariates: age, tumor subtype, pathogenic variant status, prevalent/incident BC status, and healthcare accessibility indicators.

**Results:**

Within the matched cohort, those with incident BC were significantly younger at inclusion, but were of comparable age at the time of first diagnosis. They had smaller tumors, and the odds of advanced-stage disease were approximately 30% lower than those observed in women with prevalent BC (OR = 0.29, p < 0.01). Younger age and a triple-negative phenotype were independently associated with larger tumor size. No significant effect was shown from healthcare accessibility indicators.

**Conclusion:**

The PGO’s coordinated, person-centered approach to high genetic risk management was likely associated with earlier-stage BC detection in women with the *BRCA* PV and women at high risk without *BRCA PV*. These findings both underscore the enhanced value of person-centered surveillance programs that integrate genetic risk assessment and long-term clinical follow-up, and pave the way for further research in this area.

## 1 Introduction

Women with *BRCA1* and *BRCA2* pathogenic variants (PV) are at higher risk of breast cancer (BC) than the general population. Their cumulative lifetime risks of BC are estimated at 72% (95% CI: 65%-79%) for *BRCA1* and 69% (95% CI: 61%-77%) for *BRCA2* germline carriers (1–3). An individual’s awareness of germline PV is generally contingent on their familial history of BC. This is particularly critical in apparently sporadic cases where the initial indication of genetic predisposition is early-onset BC itself, in the absence of a family history of breast or ovarian cancer, and prior to the recommended age for population-based BC screening. Conversely, for women with BC-related pedigree information available, cancer genetics services may offer a clinical assessment of BC risk using the Breast and Ovarian Analysis of Disease Incidence and Carrier Estimation Algorithm (BOADICEA) (4, 5).

For women with a confirmed germline PV, international guidelines recommend intensified screening with both MRI and mammogram for early-stage detection (6–9). Risk-reducing breast surgery is the only risk-reducing option available in France (10). However, the decision to undergo such surgery requires age-specific peer support and psychological counseling to ensure that medical recommendations align with the patient’s values and preferences (11, 12).

In France, it is recommended that PV carriers undergo intensified screening, consisting of an annual MRI scan and mammogram, as well as a clinical breast examination every six months, from ages 30 to 65, and just an annual mammogram after age 65 (10, 13). Despite the recommendations, it is possible that women’s awareness of their high genetic risk of BC may not necessarily translate into optimal adherence or improved clinical outcomes at the primary diagnosis of BC, even within equal-access healthcare systems (14, 15). There are several barriers to the uniform effectiveness of screening in women at high risk of BC (16), including unequal access to care, limited availability of MRI, non-individualized screening protocols, and organizational factors. The objective of surveillance programs is thus to promote adherence to screening recommendations by addressing organizational and accessibility issues (5, 17). Although the benefits of population-based BC screening are well-established (18), there is limited empirical evidence for screening in PV carriers. Although modeling studies have shown that management strategies for high-risk populations are both effective and cost-effective (19), these studies are based on the unrealistic assumption of perfect adherence (5, 20). The real-world study by Saule et al. (21) compared outcomes for BC diagnosed before and after genetic risk assessment (21) but data on BC diagnosis before and after inclusion in a structured, long-term risk management program based on genetic risk assessment remain scarce.

This study investigates the impact of a person-centered screening program on clinical outcomes in women who enrolled in this management program for a population with a high genetic risk.

## 2 Materials and methods

### 2.1 Study population

The Phare Grand Ouest program (PGO) is a coordination and risk management program for individuals at high genetic risk of different forms of cancer, including BC. It covers four regions in western France with a coordinating role over eight cancer genetics units. Eligible women include germline *BRCA1* and *BRCA2* PV carriers (*BRCA1+*, *BRCA2+*), as well as women with no BRCA PV but with an estimated lifetime risk of BC of more than 20% according to the BOADICEA (High risk without *BRCA*) (**Supplementary Text 1**) (5). The study population is composed of women enrolled in PGO, categorized based on their BC presentation and their risk management status at first diagnosis. Women with prevalent BC were those diagnosed prior to their inclusion in PGO. They may not have been undergoing intensified screening at the time of their primary diagnosis as their genetic counseling often started after diagnosis. However, another subset of women already knew their *BRCA* PV status prior to diagnosis, and their GP or gynecologists may have initiated intensified screening procedures for them. Nevertheless, their long-term adherence to screening was not managed systematically. Conversely, women with incident BC were cancer-free at inclusion in the program. At the time of their primary diagnosis, they were aware of their *BRCA* PV status and were under active management within PGO.

### 2.2 Ethical agreement

The present study does not involve human participants, as stipulated by the Reference Methodology (ie, MR-004), which was established by the French National Data Protection Authority (CNIL). The MR-004 framework finds application in “research that does not involve human subjects, studies, or evaluations”. The data concerns women aged ≥ 18 years who had provided written informed consent to be monitored in the cancer surveillance program and who did not object the re-use of their date for research purposes. On April 28, 2022, the Data Protection Officer (DPO) and the CREDO (Comité Recherche et Exploitation des DOnnées) at the data center (Eugene Marquis Cancer Center) formally consented to the reuse of the anonymized data for research and scientific journal submission (N°T-411).

### 2.3 Genetic testing

Genetic testing was carried out after obtaining informed written consent, through the cancer genetics units. Genomic DNA was extracted from peripheral blood, and germline testing for *BRCA1* and *BRCA2* pathogenic variants was performed in certified genetic testing platforms within the GGC-UNICANCER network. These procedures are in accordance with the guidelines of the INCa (French National Institute for Cancer) and UNICANCER. Gene sequencing and significant rearrangement analyses were conducted using standard methods at the time of testing.

### 2.4 PGO: a person-centered risk management and coordination program

In France, genetics services generally provide counseling and testing; however, they do not systematically offer long-term risk management following confirmation of a pathogenic variant. The PGO coordination program was developed to address the discrepancy between point-in-time diagnosis and long-term screening. The patient navigation consists of an operational continuum of care between medical imaging centers, general practitioners (GPs), gynecologists, and other clinical services. Upon enrollment or during follow-up, coordinators collect information regarding comorbidities, previous cancers, healthcare access, and personal constraints. This information is then used to update person-centered risk-adaptive screening plans. Organizational factors, including a structured reminder system, have been identified as a critical component of adherence to PGO (5). Women are encouraged to engage in regular correspondence with coordinators and to submit imaging reports, thereby creating an interactive feedback loop that fosters autonomy and assists in overcoming psychological barriers (12, 22). Evidence on person-centered care for individuals with a BRCA pathogenic variant emphasizes “the need for emotional support, empathy and respect” (23). Screening coordination within PGO is therefore not characterized by a paternalistic surveillance approach, but rather by an adaptative one. It integrates personal contexts – such as screening preferences or refusals, the availability of medical resources, and family responsibilities - into a holistic decision-making process. When medical or personal circumstances are temporarily incompatible with BC screening (e.g., pregnancy, breastfeeding, treatments for another type of cancer), coordinators can suggest postponing the screening until conditions allow it to be resumed. As a result, subsequent screening dates are rescheduled. When screening rounds have been completed, coordinators are responsible for summarizing the MRI and mammogram reports, translating from medical to lay language and outlining the next steps. Rather than anticipating that women will seek professional counsel in a passive manner, by arranging appointments with their GP, the screening coordination team employs a proactive approach. This strategy is designed to empower women and promote their autonomy in managing their personal health and well-being. For a detailed overview of the operational PGO framework, please refer to the **Supplementary Materials** (**Supplementary Text 2**).

### 2.5 Matching

We first identified all the women followed by the PGO program who developed incident BC. For each woman with incident BC, we proceeded with 1:1 matching by age at primary diagnosis to find women with prevalent BC. We also used pathogenic variant type as matching criteria. When an exact variant-type match was unavailable due to sample size constraints, we prioritized matching on age (**Supplementary Figure 1**).

### 2.6 Outcomes and explanatory variables

Data concerning population characteristics were retrieved from the PGO database. The primary study outcomes were the clinical characteristics of BC, which were collected through medical record review. The classification of cancer stage followed the ESMO guidelines for BC staging (24). The secondary outcome was overall survival from all causes of mortality. The database has been updated to include the most recent information on deaths by matching the database with MatchID, a free search engine for deceased persons based on INSEE (the French national institute of statistics and economic studies) death records. We also report potential overdiagnosis of cases (25, 26).

Among explanatory variables, to account for disparities in medical resources, such as access to GPs and the availability of medical imaging facilities in rural areas, we included two contextual variables for regression analysis: the Local Potential Accessibility (LPA) indicator, measuring accessibility to private GPs, standardized by age structure within municipalities (27), and MRI density measured as the number of MRI screenings per 200,000 inhabitants in the county of residence (28).

### 2.7 Statistical analysis

We used descriptive statistics to summarize the characteristics of the population studied, with a median and an interquartile range (IQR) for continuous variables, and counts and percentages for categorical variables. To compare the differences in clinical and histological characteristics between the incident and prevalent BC groups, we performed Kruskal-Wallis and Fisher exact tests. A *p-*value of < 0.05 was considered statistically significant. Graphical representations were used to illustrate age at primary diagnosis and tumor size in relation to prevalent/incident BC status. We conducted a survival analysis, using the Kaplan–Meier survival function for all-cause mortality in the prevalent BC population and BRCA1/2 pathogenic variant, with group differences assessed using the log-rank test.

For multivariable analyses, we used a generalized linear regression (GLM) with a log link function and gamma distribution with tumor size as the outcome variable. The following covariates were included in the analysis: age at primary diagnosis (continuous), TNBC status (Yes or No), pathogenic variant status, prevalent/incident BC status, LPA index (continuous), and MRI unit density. The same set of covariates was used in a logistic regression model with cancer stage as the outcome variable. According to the most recent literature, cancer stages 0 and I are classified as earlier stages of BC (0) whereas stages II, III, and IV are considered advanced stages of BC (1) (21, 24). All statistical analyses were performed using the STATA^®^ 19 software package.

## 3 Results

**Figure 1:**
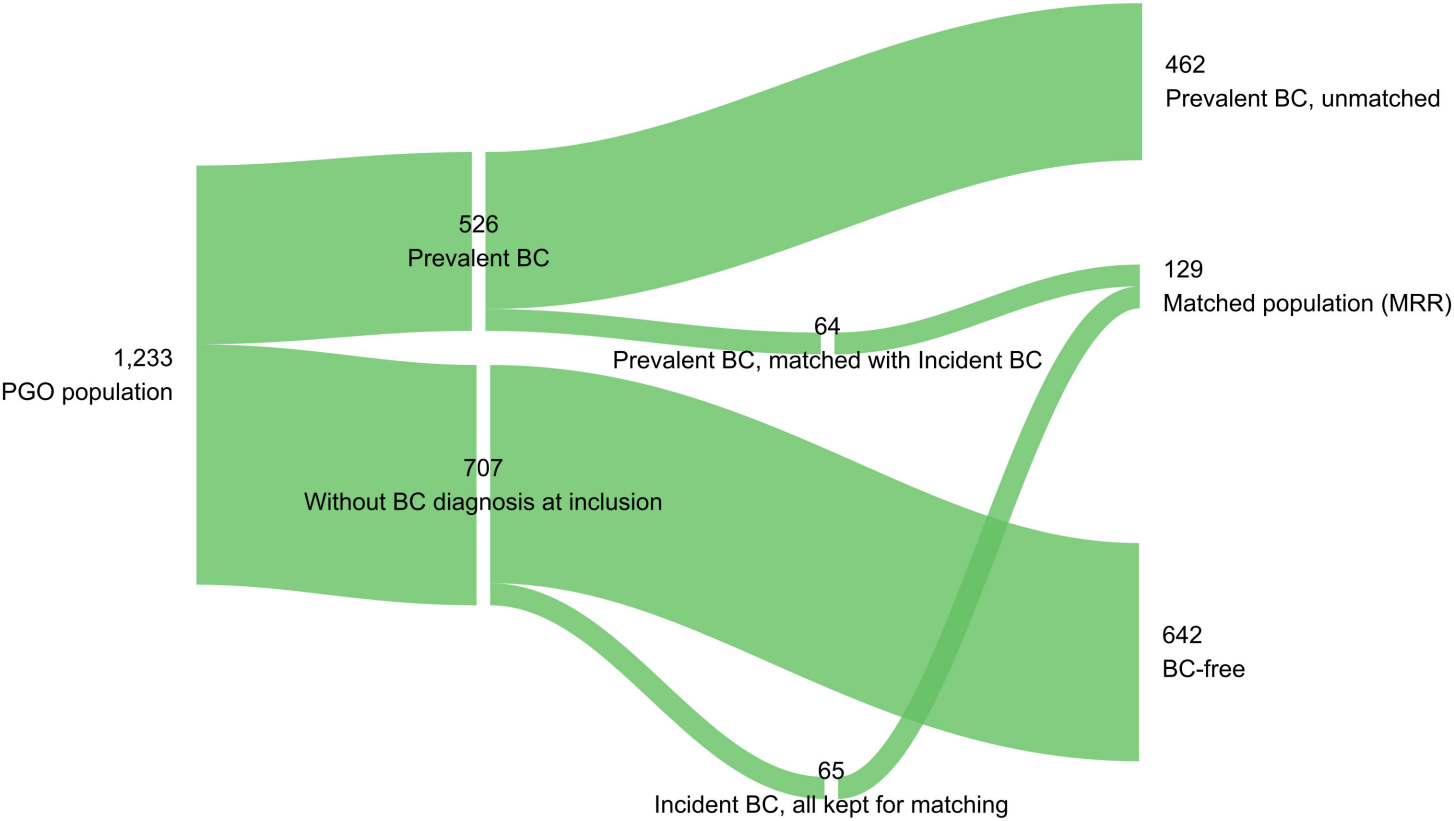
Sanky flow diagram of the study population (BC, breast cancer; MRR, medical record review).

From January 2011 to June 2022, 1,233 women were included in the PGO program. Of them, 526 (43%) had prevalent BC at inclusion, 65 (5%) developed ‘incident’ BC during follow-up and 642 (52%) were BC-free, as shown in **Figure 1**. Women with incident BC were younger (median age at inclusion 42.2, IQR 37.6—51.6) than women with prevalent BC (median age at inclusion 49.9, IQR 42.3 – 57.8) (**Supplementary Table 1**). In the matched cohort (N = 129), compared with those with prevalent BC (median age at inclusion 55.9, IQR 47.5 – 62.7) women with incident BC were younger (p<0.01), with 38% under the age of 40 (**Table 1**). Age at primary diagnosis was comparable between groups (median age at inclusion 45.4, IQR 39.3 – 56.8; 47.1, IQR 40.6 – 56.0; p=0.50), with early-onset cases (<50 years) representing 63% in both. This pattern is mirrored in **Supplementary Figure 1**, which shows the distribution of overlapping ages at primary diagnosis. The interval between primary diagnosis and inclusion in PGO was shorter for incident BC patients (median 3.3 years, IQR 1.4 – 6.6) than for prevalent BC patients (median 6.0 years, IQR 3.3 – 10.7) (**Table 1**). Pathogenic *BRCA1/2* variants were identified in 71% of the overall cohort, with a slight predominance of the *BRCA1* PV in women with incident BC (**Supplementary Table 1**); in the matched cohort. Mutational status did not differ between incident and prevalent cases (p=0.97) (**Table 2A**).

**Table 1:** Patient characteristics of the matched cohort.

	Prevalent BC		Incident BC		P-value
	N	(%)	N	(%)	
Total	64	(100)	65	(100)	
Age at inclusion (years)					
Under 40	6	(9)	25	(38)	
40-49	14	(22)	21	(32)	
50 and over	44	(69)	19	(29)	
Median (IQR)	55.9 (47.5-62.7)		42.2 (37.6-51.6)		<0.01
Pathogenic variant					
*BRCA1 +*	31	(48)	33	(51)	0.97
*BRCA2 +*	26	(41)	26	(40)	
H. risk w/o *BRCA* PV	7	(11)	6	(9)	
Age at primary diagnosis					
Under 40	18	(28)	16	(25)	
40-49	22	(34)	25	(38)	
50 and over	24	(38)	24	(37)	
Early onset BC (before 50 yrs)	40	(63)	41	(63)	
Late onset BC	24	(38)	24	(37)	
Median (IQR)	45.4 (39.3-56.8)		47.1 (40.6-56.0)		0.50
Between primary diag. and incl.					
Less than 5 years	27	(42)	42	(65)	
5-10 years	20	(31)	22	(34)	
More than 10 years	17	(27)	1	(2)	
Median (IQR)	6 (3.3-10.7)		3.3 (1.4-6.6)		
Death (All causes)					
Yes	7	(11)	1	(2)	
No	57	(89)	64	(98)	

H. risk w/o *BRCA* PV, High risk without *BRCA* PV.

**Figure 2:**
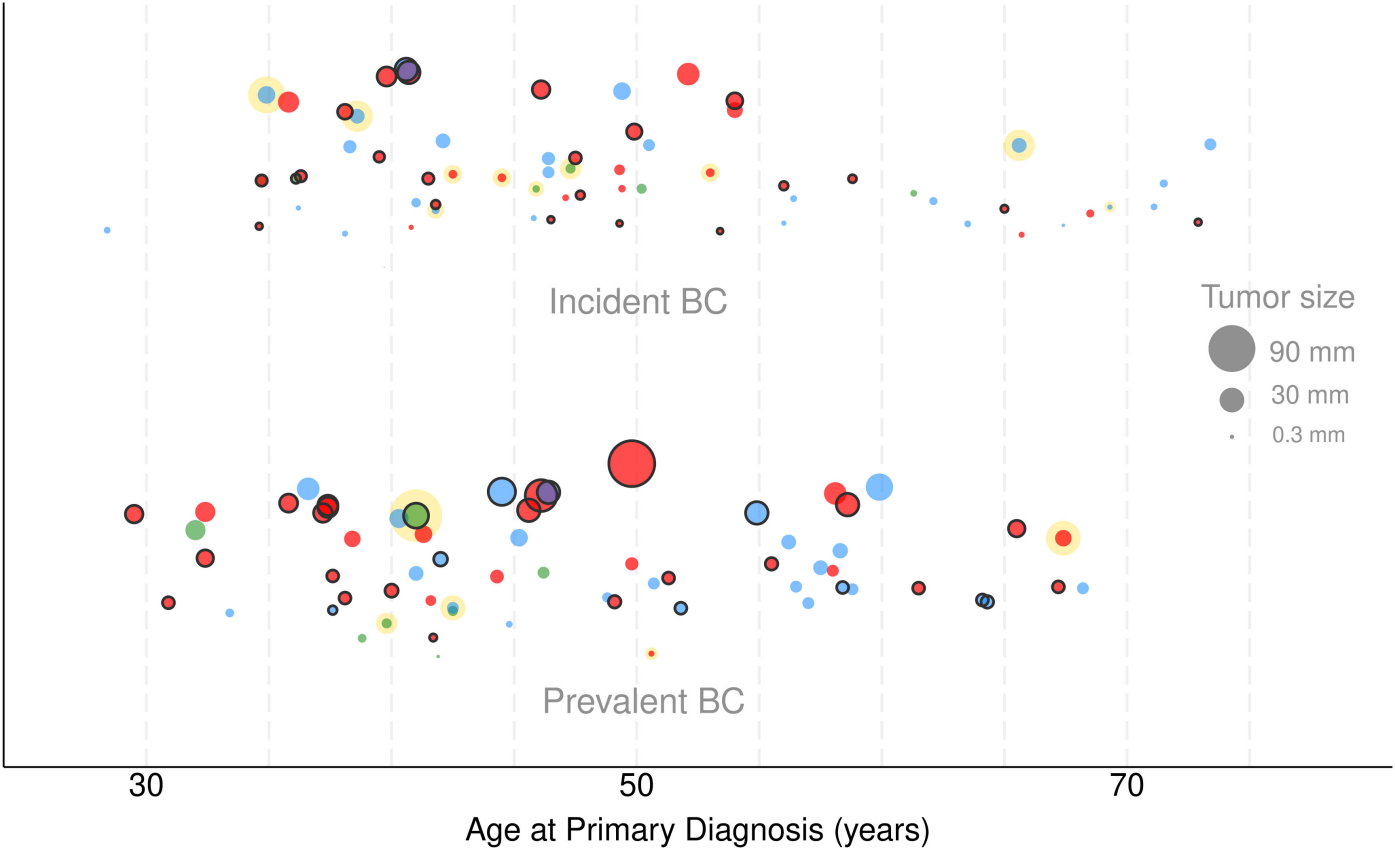
Tumor size and age at primary diagnosis, by prevalent/incident BC status. (BC, breast cancer; tumor size is represented different sized circles, applying a weighted scale; red – *BRCA1*+, blue – *BRCA2+*, green – high-risk without *BRCA* PV; black outline – triple-negative BC, without outline – HR+ or HER2+ BC; yellow halo – *in situ* BC, without halo – invasive BC).

**Figure 3:**
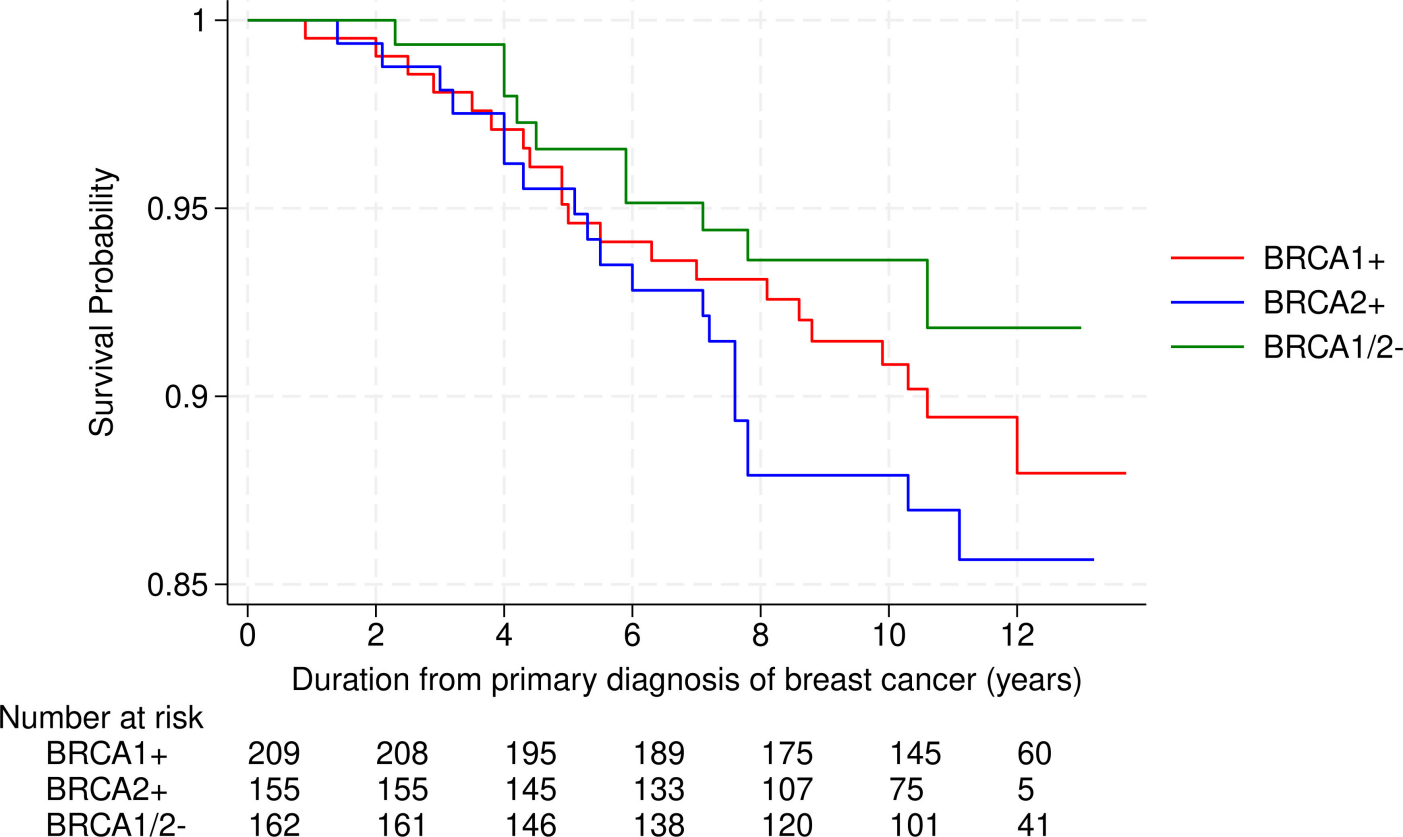
Kaplan–Meier survival curves for all-cause mortality in the prevalent BC sub-population (N = 526). H. risk w/o *BRCA* PV, High risk without *BRCA* PV.

Both the incident and prevalent BC groups had comparable histological features. Most tumors were unilateral, invasive ductal carcinomas. In terms of histological prognosis grade, there was a predominance of high-grade invasive tumors (58–67%). *In situ* lesions manifested with a slightly higher frequency in incident cases than prevalent cases (15% vs 8%), though this difference was not statistically significant (**Table 2A**). Smaller primary tumors (T1a-b) were more frequent in women with incident BC than in women with prevalent BC (58% and 10%, respectively). The frequency of node-negative disease (N0) was significantly higher in incident versus prevalent cases (p<0.001). More than three quarters of the cases of incident BC were early-stage *versus* less than half of prevalent cases (p<0.001) (**Table 2B**). This difference, shown in **Figure 2**, reflects the consistently smaller tumor size across age ranges in the incident group, compared with the prevalent group. The tumor phenotype distributions were comparable between the groups (p=0.42). Triple negative phenotypes were frequent in both prevalent and incident cases (55% and 42% respectively), while HR+/HER2- tumors were more frequent in the incident group (51% vs 36%) and HER2+ remained less frequent in both groups (**Table 2B**). **Figure 3** shows overall survival in relation to the *BRCA* PV in the prevalent BC group (N = 526). A non-significant trend for lower survival was observed in carriers of the *BRCA2* PV compared with *BRCA1* carriers and women at high-risk without *BRCA* PV (**Figure 3**).

**Table 2A:** Clinical characteristics of the matched cohort.

	Prevalent BC		Incident BC		P-value
	N	(%)	N	(%)	
Total	64		65		
Pathogenic variant					
BRCA1+	31	(48)	33	(51)	0.97
BRCA2+	26	(41)	26	(40)	
H. risk w/o *BRCA* PV	7	(11)	6	(9)	
Laterality of BC					
Right	29	(45)	28	(43)	0.99
Left	34	(53)	35	(54)	
Bilateral	1	(2)	2	(3)	
missing data	0		0		
Invas. tum. grading					
Low-grade	4	(7)	7	(13)	0.71
Intermediate	14	(26)	15	(28)	
High-grade	36	(67)	31	(58)	
missing data	10		12		
In situ tumor					
Yes	5	(8)	10	(15)	0.27
No	59	(92)	55	(85)	
In situ tummor grade					
Total	5	(100)	10	(100)	
Low-grade	0	(0)	0	(0)	0.99
Intermediate	2	(40)	4	(40)	
High-grade	3	(60)	6	(60)	
Histology					
Ductal	56	(89)	57	(88)	0.73
Lobular	1	(2)	3	(5)	
Other	6	(10)	5	(8)	

H. risk w/o *BRCA* PV, High risk without *BRCA* PV.

**Table 2B:** Clinical characteristics of the matched cohort.

	Prevalent BC		Incident BC		P-value
	N	(%)	N	(%)	
Primary tumor (T)					
Tis	5	(8)	10	(15)	
1mi	0	(0)	4	(6)	
1a	2	(3)	23	(35)	
1b	4	(7)	15	(23)	
1c	28	(47)	1	(2)	
2	20	(33)	12	(18)	
3 & 4	1	(2)	0	(0)	
missing data	4		0		
Tumor size, mm					
Median (IQR)	16 (15-30)		10 (7-17)		<0.001
missing data	4		0		
Regional lymph nodes (N)					
0	41	(67)	58	(89)	<0.001
1	20	(33)	7	(11)
missing data	3		0		
Distant metastasis (M)					
0	60	(98)	65	(100)	<0.001
1	1	(2)	0	(0)
missing data	3		0		
Cancer staging					
0	5	(8)	10	(15)	<0.001
I	24	(40)	40	(62)
II	29	(48)	15	(23)
III	1	(2)	0	(0)
IV	1	(2)	0	(0)
missing data	4		0		
Phenotype					
HER2+	5	(9)	4	(7)	0.42
HR-/HER2- (TNBC)	31	(55)	23	(42)
HR+/HER2-	20	(36)	28	(51)
missing data	8		10		
Ki 67 (cutoff 20%)					
Negative	2	(17)	6	(22)	<0.001
Positive	10	(83)	21	(78)
missing data	52		38		

**Table 3** shows the multivariable analyses of the effects of factors on tumor size and cancer stages. After adjustment for other covariables, age at primary diagnosis was inversely associated with tumor size (p=0.02). This suggests that younger age was associated with larger tumor size. TNBC was associated with tumor size (p=0.04), and women with incident BC had significantly smaller tumors (GLM model). The odds of advanced stage were 0.29 times lower than those observed in women with prevalent BC (OR = 0.29 95% CI: 0.12-0.66, p < 0.01). BRCA1 PV carriers had 15 times greater odds of being diagnosed with advanced-stage BC than women at high-risk without *BRCA* PV (OR = 14.92, 95% CI: 1.60 to 138.90, p = 0.02). No significant associations were found for *BRCA2* PV status and age. Similarly, there was no significant effect for the factor accessibility to GPs and MRI units. Two women aged over 65 years in the incident group were diagnosed with carcinoma *in situ*, and these may be over-diagnosed cases (**Figure 2**).

**Table 3:** Multivariable analysis.

Covariable	Tumor size (Generalized linear model)				Cancer stage (Logistic regression)			
	Coefficient (95% CI)	SE	z	p-value	Coefficient (95% CI)	SE	z	p-value
Age at primary diagnosis (continuous)	-0.01 (-0.02 to -0.02)	0.01	-2.34	0.02	0.97 (0.93 to 1.01)	0.02	-1.53	0.13
Triple negative breast cancer								
Yes	0.24 (0.01 to 0.47)	0.12	1.00	0.04	1.58 (0.65 to 3.82)	0.71	1.02	0.31
No (Reference)	–	–	–					
Mutation status								
BRCA1+	0.26 (-0.12 to 0.63)	0.19	1.35	0.18	14.93 (1.61 to 138.90)	16.99	2.38	0.02
BRCA2+	0.27 (-0.10 to 0.64)	0.19	1.43	0.15	8.24 (0.89 to 75.93)	9.34	1.86	0.06
H. risk w/o *BRCA* PV (Ref)								
Prevalent / Incident BC status								
Incident BC	-0.48 (-0.69 to -0.27)	0.11	-4.51	<0.001	0.29 (0.13 to 0.66)	0.12	-2.94	<0.01
Prevalent BC (Reference)	–	–	–		–	–	–	
Local potential accessibility (continuous)	0.09 (-0.02 to 0.20)	0.06	1.53	0.13	1.10 (0.73 to 1.69)	0.24	0.44	0.66
MRI units per 200,000 inhabitants (continuous)	0.03 (-0.11 to 0.17)	0.07	0.42	0.68	1.05 (0.59 to 1.87)	0.31	0.17	0.87
Intercept	2.94 (2.26 to 3.61)	0.35	8.49	<0.001	0.26 (0.01 to 6.95)	0.44	-0.80	0.43

H. risk w/o *BRCA* PV, High risk without *BRCA* PV.

## 4 Discussion

The aim of this study was to provide empirical evidence of whether BC diagnosed during follow-up by a coordinated risk management program differed from BC diagnosed without being in the program. We analyzed real-world data on women at high risk of BC confirmed by oncogenetic counseling and genetic testing. We found significantly smaller tumor sizes and lower cancer stages at primary diagnosis in BC cases that were diagnosed during the program’s follow-up period. These findings suggest that this type of risk management program, based on genetic testing, is likely to be clinically effective in ensuring early detection of BC related to pathogenic mutations.

To account for the fact that women with prevalent BC were younger at BC onset than those with incident BC, we matched women on their age at primary diagnosis prior to reviewing medical records. In the matched cohort, women with incident BC were younger at inclusion than those with prevalent BC, a finding which is consistent with the slightly higher proportion of *BRCA1* PV carriers and the earlier onset of the tumors related to this. The incident cases were diagnosed more often at stage 0-1, with smaller tumor size, in contrast to prevalent BC cases which at more advanced stages. This result was statistically significant and consistent across univariable and multivariable analyses. Independently of the clinical factors influencing cancer stage, including age at primary diagnosis, triple-negative breast cancer status, and pathogenic variant type, it was found that participating in the risk-management program was likely to promote earlier cancer detection. Furthermore, the contextual determinants that are susceptible of affecting early detection of BC and that were adjusted for by the model, such as indicators for healthcare accessibility, did not result in a statistically significant association with tumor size or cancer stage. In terms of clinical characteristics, most BC cases were unilateral and invasive ductal cancers with only a minority classified as HER2+ cases, but no statistically significant differences were observed between incident and prevalent cases. This may partly be explained by the relatively small number of cases within subgroups. We expected that women who were *BRCA1* carriers would have more aggressive BC due to the higher prevalence of triple-negative tumors (29). This was not found in our study. Despite the limited sample size, our findings revealed twice as many cases of ductal carcinoma *in situ* in the incident BC group compared to the prevalent group, classified as a very early form of BC (25). The results of this study reveal a noteworthy pattern in women with prevalent BC who, as already mentioned, were younger at the time of primary diagnosis compared with those with incident BC. This likely reflects the fact that the early-onset BC in women without a family history of BC is often the first indication of their underlying genetic predisposition.

Previous studies have examined the clinical characteristics of BC in *BRCA1*/*2* carriers through two key comparisons: screen-detected *versus* interval cancer cases, and cancers diagnosed before *versus* after genetic testing (13, 21). These studies reported comparable evidence of a lower cancer stage associated with early detection, as was observed in the present study, where incident cases were diagnosed more often at stage 0-1, with smaller tumor size. This outcome differs from matched prevalent BC cases which exhibited more advanced stages of the disease. Our study contributes novel insights to the extant body of work in this field. First, our research focused on the specific impact of a coordinated risk management program. Previous studies delineated the comparison groups through the classification of participants as either those who underwent genetic testing in conjunction with intensive screening or those who did not undergo such testing (21, 30). The present analysis shifts the focus to a subsequent phase by conducting a comparison of outcomes with *versus* without being followed by a risk management program that creates a person-centered continuum of care. The positive clinical outcomes observed in the PGO program are most likely indicative of its person-centered coordination model. In addition, our study provides real-world evidence consistent with findings in the general population, in which adherence to national screening recommendations has been associated with BC diagnosed at earlier clinical stages (18). This highlights how empirical data are of outmost interest in complementing existing cost-effectiveness modeling (19). In the incident BC group, two women aged over 65 years and who were diagnosed with carcinoma *in situ* may have been over-diagnosed (25, 26). The gold standard for assessing overdiagnosis is to conduct a randomized controlled trial comparing the cumulative incidence in screened and unscreened groups, which would be interesting to do in future studies. Despite the increased costs and disutility associated with overdiagnosis, past cost-effectiveness analyses of intensified screening programs did not take this aspect into account (16, 19, 25, 26, 30). Future cost-effectiveness analysis of the PGO could integrate these inputs to provide a complete assessment of the value of the program.

Our study has several limitations. It relies on the size of our sample groups, which reflects the absence of a single national program in France for a high genetic risk of BC in contrast to the one for BC screening in the general population. In France, there are 17 programs at a regional or inter-regional level for this population at very high risk of BC. We also note the possible heterogeneity in high-risk women without *BRCA* PV as the risk of BC was estimated using the BOADICEA model, which was not updated with environmental factors or the polygenic risk score (PRS). However, Bhatt et al. investigated how PRS and other risk factors could be associated with the progression of BC and found little difference in the sojourn time between low-risk and high-risk based on PRS (4). Due to certain inherent features of both the high-risk population and the program itself, we recommend that future studies evaluate the program’s effectiveness by using a more holistic approach. For instance, qualitative research on women’s perception could be conducted. PGO was conceptualized as a person-centered risk management program designed for implementation in cancer genetics units that announce genetic results. To evaluate the various stages that result from knowledge of genetic results (i.e. patient’s values and choices with regard to autonomy, adherence to screening, support throughout the long follow-up care journey), it would be worthwhile having both qualitative and quantitative indicators available. Finally, we used area-based variables to measure patients’ socioeconomic and other contextual factors. A better approach would be to use an address-based deprivation index to minimize the biases in the estimates (31, 32).

Beyond outcomes, one significant strength of our study is its extension of existing evidence regarding the effects of screening in women with the BC *BRCA1* and *BRCA2* PV. There is a lack of empirical data for this demographic. The present analysis offers a comprehensive update and evaluation of one of the French regional high-risk genetic screening programs. The findings of this study could contribute to more nuanced understanding of the clinical and genetic profiles associated with BC detected under structured and person-centered risk screening programs. This understanding could facilitate the development of optimized follow-up strategies for women with a high genetic risk of BC. Furthermore, our findings highlight the value of integrating routinely collected program data for refining screening recommendations and better estimating the true benefits of high-risk surveillance in practice (5, 20).

In conclusion, this analysis of the PGO population highlights the clinical and genetic characteristics of BC occurring under structured surveillance in comparison with those diagnosed prior to joining the program. The study provides novel insight into breast cancers detected through person-centered risk-management programs and supports further prospective evaluation of their effectiveness and sustainability in the prevention of high genetic risk of BC.

## Data Availability

Publicly available datasets were analyzed in this study. This data can be found here: https://doi.org/10.5281/zenodo.17937993.
