# Supplementary material for: From genetic risk to early detection - clinical outcomes of a person-centered screening program for women with a high genetic risk of breast cancer
Source: Front Oncol. 2026 Feb 2;15:1730423. doi: 10.3389/fonc.2025.1730423 (PMC12907759; doi:10.3389/fonc.2025.1730423)
Supplement: Supplementary file 1 [file DataSheet1.docx]

From Genetic Risk to Early Detection - Clinical Outcomes of a Person-Centered Screening Program for Women with a High Genetic Risk of Breast Cancer

Ke ZHOU^1*,^ Caroline Abadie^2^, Louise Crivelli^3^, Euriell Fortin^4^, Martine Bellanger^1^, Charlotte Huet^4^

^1^Departement of Human and Social Sciences, Institut de Cancérologie de l’Ouest, F 44805 Saint Herblain, France

^2^Departement of Oncogenetics, Institut de Cancérologie de l’Ouest, F 44805 Saint Herblain, France

^3^Departement of Oncogenetics, Centre Eugène Marquis, F 35000 Rennes, France

^4^Coordination of the Phare Grand Ouest, Centre Eugène Marquis, F 35000 Rennes, France

*** Correspondence:**Corresponding Authors
Ke.Zhou@ico.unicancer.fr
Martine.Bellanger@ico.unicancer.fr

**SUPPLEMENTARY MATERIALS**

Supplementary Text 1

Women included in PGO carry a BRCA1 or BRCA2 pathogenic variant with no prophylactic bilateral mastectomy and no progressive cancer.

PGO is also offered to women with no BRCA pathogenic variant (PV) but with a high-risk for BC, defined as an estimated lifetime BC risk >20% calculated using the Breast and Ovarian Analysis of Disease Incidence and Carrier Estimation Algorithm— Boadicea-model. In our analyses, these women were in the group "High risk without BRCA PV". According to French guidelines, women with no identified PV are also considered to be at high risk of BC if their lifetime BC risk is more than 20-25%.

Since PGO was initiated in 2011, the Boadicea versions used have not included non-familial BC risk factors such as breast density, reproductive, and lifestyle patterns.

Supplementary Text 2

| **The Phare Grand Ouest – PGO- Program and its operational reminder system**  Coordinating how patients navigate an operational continuum of care between medical imaging centers, general practitioners (GPs), gynecologists, and other clinical services is facilitated by dedicated coordinators. At inclusion or during subsequent follow-up, comprehensive assessments are conducted to collect information regarding the presence of comorbidities, cancers other than BC, access to health care services, availability of imaging facilities, and personal constraints such as the responsibility of caring for family members. This information is then used to generate a personalized, risk-adaptive screening plan, incorporating a structured reminder system.  Routine reminder letters are sent three months prior to the scheduled examination date, with an additional notice two months later, if the examination has not yet been completed. When a radiologist requests a recall, coordinators verify through reminders that a new appointment has been arranged.  Women are encouraged to correspond directly with the coordination team and to submit imaging reports, thereby creating an interactive feedback loop that helps overcome psychological barriers such as forgetfulness, procrastination, and anxiety related to MRI procedures, including claustrophobia and intravenous line placement as well as overall stress (1).  To facilitate access to screening, the coordination team issues formal requests to GPs or gynecologists, or “priority letters” to radiologists. Each participant is also issued a priority identification card to make possible efficient transmission of imaging reports to the PGO team. These organizational measures have been shown to minimize administrative barriers that could otherwise reduce compliance with recommended screening protocols, as well as alleviate stress associated with scheduling or care delays (2).  According to recommendations, combined imaging with mammogram and MRI is advised to reduce interval cancers (3). However, some women may undergo either MRI or mammogram. In such cases, program coordinators request that both examinations be completed. For women with no identified *BRCA* variant but with a high risk of BC, renewed genetic counseling is proposed every 10 years.  1. Berg WA, Bandos AI, Sava MG. Analytic Hierarchy Process Analysis of Patient Preferences for Contrast-Enhanced Mammography Versus MRI as Supplemental Screening Options for Breast Cancer. Journal of the American College of Radiology (2023) 20:758–768. doi: 10.1016/j.jacr.2023.05.014  2. Johnsen KB, Strømsvik N. Need for specially designed educational support groups: Young women’s experiences of being identified with BRCA pathogenic variants. Journal of Genetic Counseling (2025) 34:e1980. doi: 10.1002/jgc4.1980  3. Pilewskie M, Zabor EC, Gilbert E, Stempel M, Petruolo O, Mangino D, Robson M, Jochelson MS. Differences between screen-detected and interval breast cancers among BRCA mutation carriers. Breast Cancer Res Treat (2019) 175:141–148. doi: 10.1007/s10549-018-05123-6 |
| --- |

Supplementary Figure 1 Age at primary diagnosis of breast cancer in the PGO population (A), and matched cohort (B) (BC – breast cancer)

| 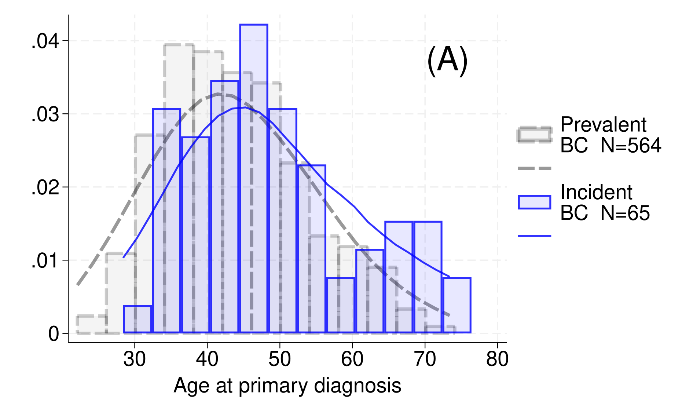 | 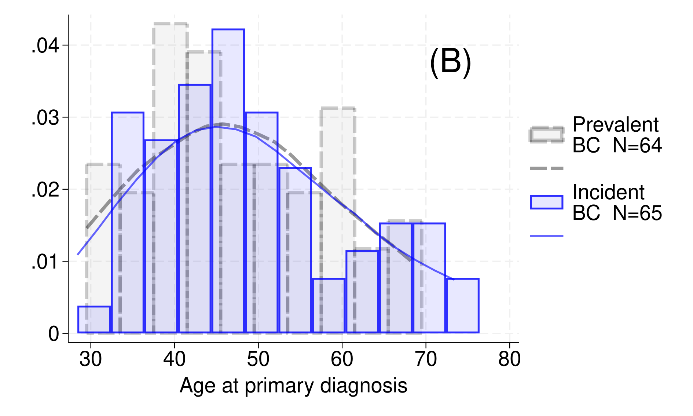 |
| --- | --- |

Supplementary Table 1 Patient characteristics of the PGO population (N=1233)

|  | Prevalent BC |  | Incident BC |  | BC-free |  |
| --- | --- | --- | --- | --- | --- | --- |
|  | N | (%) | N | (%) | N | (%) |
| Total | 526 | (100) | 65 | (100) | 642 | (100) |
| Age at inclusion (years) |  |  |  |  |  |  |
| Under 40 | 94 | (18) | 25 | (38) | 263 | (41) |
| 40-49 | 170 | (32) | 21 | (32) | 178 | (28) |
| 50 and over | 262 | (50) | 19 | (29) | 201 | (31) |
| Median (IQR) | 49.9 (42.3-57.8) | | 42.2 (37.6-51.6) | | 43.9 (35.2-54.2) | |
| Pathogenic variant |  |  |  |  |  |  |
| *BRCA1 +* | 209 | (40) | 33 | (51) | 262 | (41) |
| *BRCA2 +* | 162 | (31) | 26 | (40) | 220 | (34) |
| *BRCA1/2 -* | 155 | (29) | 6 | (9) | 160 | (25) |
| Death (All causes) |  |  |  |  |  |  |
| Yes | 51 | (10) | 1 | (2) | 32 | (5) |
| No | 476 | (90) | 64 | (98) | 608 | (95) |
| Age at death |  |  |  |  |  |  |
| Total | 51 | (100) | 1 | (100) | 32 | (100) |
| Under 50 | 13 | (25) | 0 | (0) | 2 | (6) |
| 50-59 | 10 | (20) | 0 | (0) | 4 | (13) |
| 60 and over | 28 | (55) | 1 | (100) | 26 | (81) |
| Median (IQR) | 62.1 (48.4-70.5) | | 70.1 (70.1-70.1) | | 68.6 (61.7-76.5) | |
